# XRN2 Links RNA:DNA Hybrid Resolution to Double Strand Break Repair Pathway Choice

**DOI:** 10.3390/cancers12071821

**Published:** 2020-07-07

**Authors:** Tuyen T. Dang, Julio C. Morales

**Affiliations:** 1 Department of Neurosurgery, University of Oklahoma Health Science Center, Oklahoma City, OK 73104, USA; Tuyen-Dang@ouhsc.edu; 2 Department of Neurosurgery, Stephenson Cancer Center, University of Oklahoma Health Science Center, Oklahoma City, OK 73104, USA

**Keywords:** RNA:DNA hybrids, non-homologous end-joining, homologous recombination

## Abstract

It was recently shown that the 5’ to 3’ exoribonuclease XRN2 is involved in the DNA damage response. Importantly, loss of XRN2 abrogates DNA double stranded break repair via the non-homologous end-joining pathway. However, the mechanistic details of how XRN2 functions in the non-homologous end-joining repair process are unknown. In this study, we elucidated that XRN2-mediated RNA:DNA hybrid resolution is required to allow Ku70 binding to DNA ends. These data suggest that XRN2 is required for the initiation of non-homologous end-joining repair. Interestingly, we uncovered a role for XRN2 in the homologous recombination repair pathway. Loss of XRN2 lead to a decrease in the repair of double strand breaks by homologous recombination. Strikingly, when we removed RNA:DNA hybrids by RNaseH1 over-expression, homologous recombination was not restored. We found RNA:DNA hybrid formation at and downstream of the DSB site, suggesting that unregulated transcription inhibits homologous recombination repair. In summary, our results indicate a relation between RNA:DNA hybrid resolution and double strand break repair pathway choice.

## 1. Introduction

Maintaining genomic integrity over multiple rounds of replications is essential for cellular survival. The cell encounters a variety of different types of genomic lesions on a daily basis, and the most dangerous lesion is a DNA double stranded break (DSB), as one unrepaired DSB can be detrimental to cellular survival [1]. This DSB will initiate a signaling cascade known as the DNA damage response (DDR) [1] Unrepaired DSBs are also dangerous due to the potential of the free DNA end to invade a neighboring DNA strand thus resulting in translocations which can contribute to disease states such as cancer [1]. To overcome the threat of DSBs, the cell employs one of two major DSB repair pathways: non-homologous end-joining (NHEJ) or homologous recombination (HR) [2].

The decision on whether to use the HR or NHEJ repair pathway is extremely important as NHEJ repair is an “error-prone” process and highly susceptible to introducing mutations due to loss of genetic material at the break site and HR is an “error-free” process that restores any potentially lost genetic information through the use of a sister chromatid [3]. How the decision is made between these two pathways is not clearly understood.

One process that complicates DNA repair choice is transcription [4]. It is commonly observed that highly transcribed areas of the genome preferentially employ the HR repair pathway [4], while transcriptionally silent areas of the genome tend to elicit the NHEJ repair pathway [5]. How transcription affects DNA repair pathway choice is not known.

A consequence of transcription is the formation of an extended RNA:DNA hybrid or R loop [6]. There are several lines of evidence that suggest R-loop formation in the genome leads to DSB formation [7,8,9,10]. Yet, it appears that RNA:DNA hybrids not only contribute to genomic instability, but are required for proper DSB repair [9,10,11]. How R loops contribute to DNA repair and DNA repair pathway choice requires further investigation. 

One cellular factor that connects the DDR to R loops is XRN2 [8]. XRN2 is a 5’–3’ exoribonuclease that is conserved in eukaryotic cells. XRN2 is a nuclear protein that recognizes the 5’ mono-phosphate of single stranded RNAs and degrades them to mononucleotides [12]. One critical function of XRN2 is RNA polymerase II (RNAPII) displacement at the 3’ end of coding genes [12,13]. Our previous study also identified XRN2 as a novel DNA repair factor [8]. We demonstrated that XRN2 is required for genomic stability and resistance to genotoxic stress. Yet, the mechanistic details of how XRN2 functions in DNA repair are still undetermined.

In this study, we find that loss of XRN2 inhibits Ku70 and RPA32 from binding DNA ends. Concurrently, we discovered an extended association of EXO1 with chromatin, suggesting that the defective HR repair observed after XRN2 loss is due to extended DNA resection. We also observed RNA:DNA hybrid formation both up- and downstream of DSBs after XRN2 loss. In total, our data suggest that displacement of RNAPII from the DNA template in a timely manner is a contributing factor in proper DNA repair.

## 2. Results

### 2.1. Loss of XRN2 Abrogates Ku70 Binding at the DSB Repair Site

**Figure 1:**
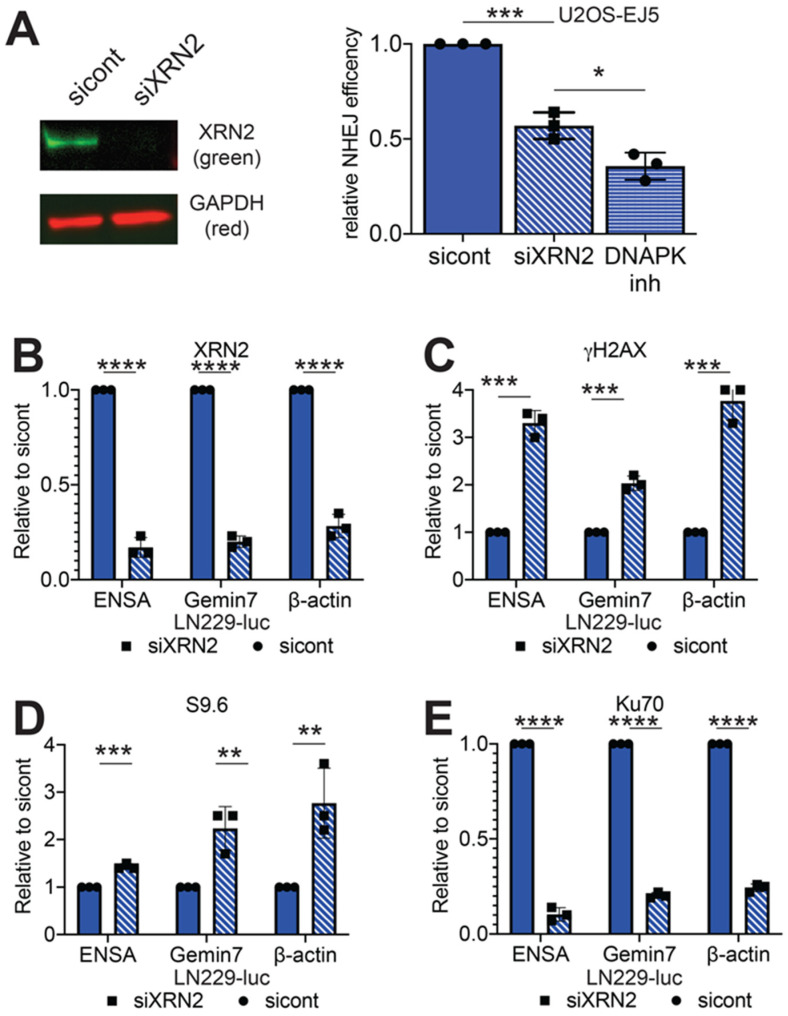
XRN2 loss decreases Ku70 binding to chromatin. (**A**) Steady-state protein levels of XRN2 were interrogated in the NHEJ U2OS-EJ5 reporter cells exposed to control or XRN2 siRNAs and NHEJ repair in the U2OS-EJ5-reporter cells was determined by measuring 1000 cells exposed to control siRNAs, XRN2 siRNAs or 100 nM NU7026. Chromatin immunoprecipitation/qPCR (CHIP/qPCR) experiments were performed using (**B**) XRN2, (**C**) γH2AX, (**D**) S9.6 and (**E**) Ku70 antibodies in LN229-luc cells exposed to control or XRN2 siRNAs at the 3’ pause sites of the *ENSA*, *Gemin7* and *β-actin* genes. Statistical analysis was done using Student’s *t*-test. * = *p* < 0.05, ** = *p* < 0.01, *** = *p* < 0.0001 and **** = *p* < 0.00001. Detailed information about Western blot can be found at Figure S9.

Previously, we demonstrated that XRN2 forms distinct nuclear foci, colocalizing with RNA:DNA hybrids in response to ionizing radiation (IR) exposure [8]. XRN2 also colocalizes with 53BP1, a DSB marker after IR (0.5 Gy) (Figure S1). Cells lacking XRN2 demonstrate increased sensitivity to IR [8]. This increased sensitivity to IR often arises when DSB repair pathways are defective. DSBs are typically repaired by the HR or NHEJ repair pathways [1]. NHEJ can be further broken up into two distinct pathways: classical NHEJ (cNHEJ) or alternative NHEJ (aNHEJ) [14]. Loss of XRN2 impedes the repair of linearized DNA plasmids by the NHEJ pathway [8]. To ensure this loss of NHEJ repair was not an artifact of a plasmid-based system, we examined how loss of XRN2 affects NHEJ on a chromosomal substrate. We exposed U2OS-EJ5 NHEJ reporter cells [15] to control or a pool of three non-overlapping XRN2 siRNAs (Figure 1A). The EJ5 reporter cells measure total end-joining events; cNHEJ and aNHEJ events and contain an expression cassette for green fluorescent protein (GFP) that is interrupted by an I-SceI endonuclease restriction site. Expression of the I-SceI endonuclease generates a DSB, which when repaired accurately leads to a GFP expression. Consistent with our previous data, loss of XRN2 led to a ~50% decrease in GFP expressing cells as compared to control cells (Figure 1A). This ~50% decrease in GFP expressing cells after XRN2 loss was similar but not quite to same degree as the ~65% decrease observed when the EJ5 cells were treated with 100 nM of NU7026, a DNAPK small molecule inhibitor [16] (Figure 1A). Interestingly, loss of XRN2 resulted in a decrease of spontaneous DNAPKcs threonine 2609 phosphorylation (Figure S2), a DNAPKcs activation mark [17].

One of the first steps in the NHEJ pathway is binding of the Ku70/Ku80 heterodimer to the DSB site, a step that is required for activation of the DNAPKcs subunit [3]. To determine if there was a defect in Ku70 recruitment to a DSB site after XRN2 loss, we coupled chromatin immunoprecipitation with qPCR (ChIP/qPCR). We examined the 3’ pause sites of the *β-actin*, *ENSA* and *Gemin7* genes, which undergo R-loop dependent transcription termination and have been previously shown to accumulate DNA damage response proteins with loss of XRN2 [8]. We treated LN229-luc cells with control or a pool of three non-overlapping XRN2 siRNAs (Figure S3). Importantly, consistent with previously published data [9], we found no change in the cell cycle distribution between cells with and without XRN2 (Figure S4). We detected an 86, 83 and 73 percent reduction in XRN2 signal when we performed ChIP/qPCR experiments using a XRN2 antibody at the 3’ pause sites of the *ENSA*, *Gemin7* and *β-actin* genes, respectively; confirming XRN2 loss (Figure 1B). In addition, depletion of XRN2 lead to a 3.0-, 2.0- and 3.8-fold increase qPCR signal in γH2AX pulldowns (Figure 1C), as compared to control cells at each of the loci tested. Furthermore, a 1.4-, 2.2- and 2.8-fold increase in RNA:DNA hybrids was observed, when using the S9.6 antibody, an antibody that primarily recognizes RNA:DNA hybrids [18] (Figure 1D). These increases in S9.6 and γH2AX at these loci are consistent with previously published data [8,9]. However, when an antibody for Ku70 was used to perform similar experiments, there was a 93, 81 and 76 percent reduction in signal at all three genomic loci (Figure 1E). This decrease of Ku70 signal at the 3’ pause region of the *β-actin* gene was also seen in LN229 or G55 human glioblastoma cells infected with a lentivirus expressing a XRN2 shRNA (LN229-shXRN2 or G55-shXRN2) as compared to LN229 and G55 cells expressing a luciferase shRNA (Figure S5). These data suggest that loss of XRN2 leads to a defect in the initial steps of the cNHEJ pathway. Namely, while loss of XRN2 led to DSB formation, as indicated by an increase in γH2AX signal at the 3’ end of the genes examined, Ku70 recruitment to these sites was inhibited.

Surprisingly, loss of XRN2 has no effect on aNHEJ. We obtained the U2OS-EJ2 reporter cells that specifically measures aNHEJ [15]. Similar to the EJ5 reporter cell line, the EJ2 cells contain a GFP expression cassette interrupted by an I-SceI endonuclease site. This I-SceI generated DSB is designed to be repaired by the aNHEJ pathway. We transfected EJ2 cells with control or XRN2 siRNAs and found no difference in GFP expressing cells between EJ2 cells with and without XRN2 (Figure S6A), suggesting that loss of XRN2 has no effect on the aNHEJ repair pathway. The aNHEJ pathway requires a functional PARP1 [19]. In addition to no defect in aNHEJ repair, loss of XRN2 also decreased the colony forming ability of LN229-shXRN2 cells as compared to control cells, when exposed to Niraparib; a PARP1 small molecule inhibitor [20] (Figure S6B). Consistent with these data, XRN2 loss leads to an increase in parylated PARP1, an active form of PARP1 [21], while total PARP1 levels remain unchanged (Figure S6C). PARP1 parylation in XRN2 deficient cells is similar to that of cells that have lost RPRD1B [22]. These data suggest that loss of XRN2 activates and increases the cells reliance of PARP1 for survival.

### 2.2. Loss of XRN2 Results in Impaired HR Repair

Loss of XRN2 results in an increase in chromatid type breaks, a characteristic found in cells with defective HR repair [8]. In addition, LN229-shXRN2 demonstrate increased sensitivity to the PARP1 inhibitor, Niraparib, as compared to control cells (Figure S6B). Increased cellular sensitivity to PARP1 inhibition is a common trait found in cells impaired in HR repair [23]. These data suggest that loss of XRN2 negatively affects the HR repair pathway.

**Figure 2:**
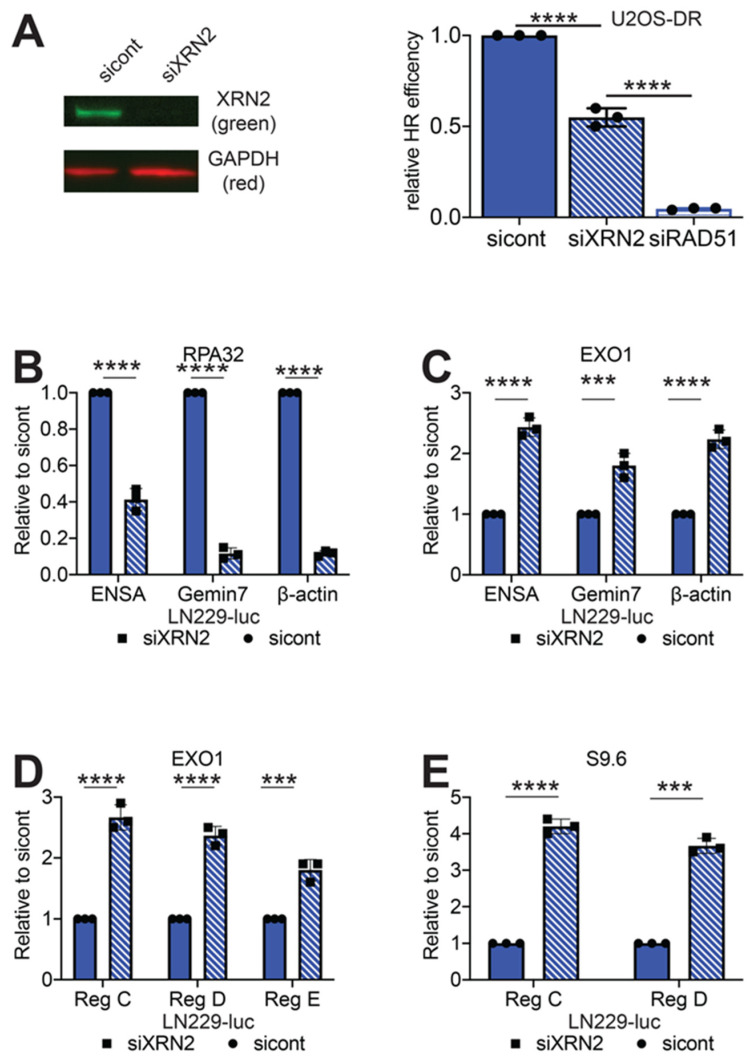
Loss of XRN2 disrupts HR repair. (**A**) Steady state protein levels of XRN2 was measured by Western blot in U2OS-DR reporter cells treated with control or XRN2 siRNAs and efficiency of HR repair in the U2OS-DR cells exposed to control, XRN2 or RAD51 siRNAs and determined by measuring 1000 cells for GFP expression. Chromatin immunoprecipitation/qPCR experiments were performed using (**B**) RPA32, (**C**,**D**) EXO1 and (**E**) S9.6 antibodies in LN229-luc cells exposed to control or XRN2 siRNAs at the 3’ pause site, Region C (Reg C) Region D (Reg D) and Region E (Reg E). Statistical analysis was done using Student’s *t*-test. **** = *p* < 0.0001 and *** = *p* < 0.001. Detailed information about Western blot can be found at Figure S9.

To study the possible role of XRN2 in the HR repair pathway, we employed U2OS-DR reporter cell line. Similar to the EJ2 and EJ5 reporter cell lines, DR cells express GFP after an I-SceI generated DSB has been repaired via HR [24]. We exposed the DR cells to control or XRN2 siRNAs. We found a 50% decrease in recombination repair efficiency in the XRN2 deficient cells as compared to the control cells (Figure 2A). Yet, this 50% decrease in GFP expressing cells is not to the same extent of the 95% decrease observed when DR cells are treated with RAD51 siRNA, suggesting that while important, XRN2 is not an essential homology-driven repair event.

### 2.3. Loss of XRN2 Results in an Extended Association of EXO1 with Chromatin

Loss of XRN2 leads to a decrease in HR repair efficiency (Figure 2A) and increased RNA:DNA hybrid formation at each of the 3’ pause sites of the *ENSA*, *Gemin7* and *β-actin* genes (Figure 1D). As it has been previously shown that RNA:DNA hybrid formation impairs recruitment of the RPA complex, an important step in HR repair, to the DSB site [11,25], we examined whether loss of XRN2 affected RPA complex formation at the 3’ ends of the *ENSA*, *Gemin7* and *β-actin* genes. Using an antibody to RPA32, one of the subunits of the RPA complex, to perform ChIP/qPCR, we observed 60, 89 and 88 percent decreases in RPA32 signal at each of the three genomic loci tested (Figure 2B). Interestingly, we found 2.4-, 1.8- and 2.1-fold increases in EXO1 signal at each of the loci tested (Figure 2C). These data are consistent with our previous observation that loss of XRN2 results in increased CtIP accumulation at the 3’ pause sites of the *ENSA*, *Gemin7* and *β-actin* genes [8] and an increase in spontaneous MRE11 foci formation (Figure S7). In addition to the 3’ pause site of the *β-actin* gene, we examined regions up- and downstream of this site for EXO1 and RNA:DNA hybrid accumulation. For these experiments, we used primer sets provided by Manley and colleagues for regions C, D and E [13], which represents roughly -2000, +250 and +1000 nucleotides, respectively, in relation to the 3’ pause site of the *β-actin* gene. With loss of XRN2, we found a 2.6-, 2.4- and 1.8-fold increase in EXO1 signal after ChIP/qPCR as compared to control cells, at regions C, D and E, respectively (Figure 2D). In addition, we found a 4.2- and 3.7-fold increase in S9.6 signal at regions C and D after XRN2 loss (Figure 2E). The extended association of EXO1 with chromatin after XRN2 loss is suggestive of increased long-range DNA degradation, which can inhibit HR repair [26] and is facilitated by transcription.

### 2.4. RNaseH1 Expression Restores NHEJ Repair

**Figure 3:**
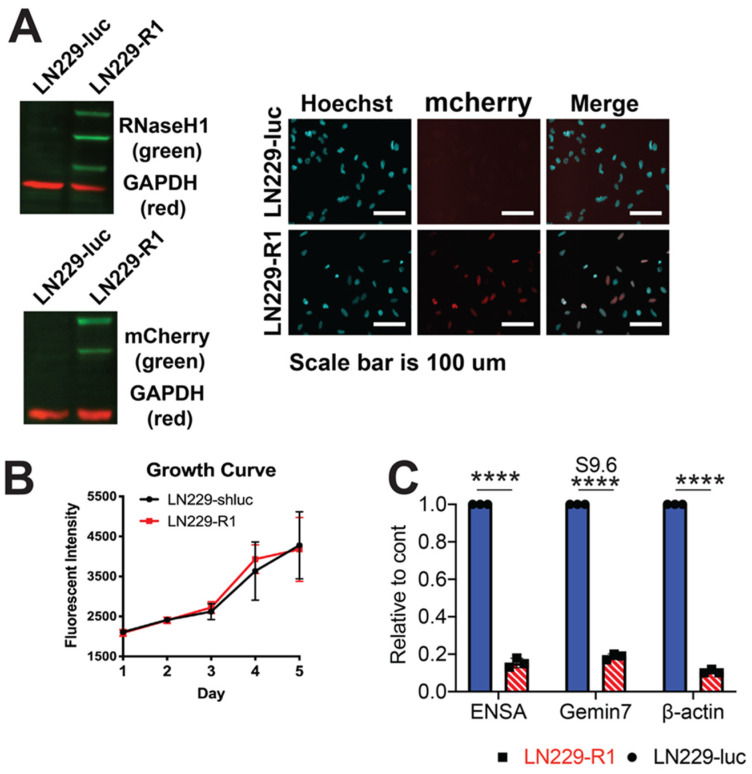
Human RNaseH1 can be stably expressed and is functional in LN229 glioblastoma cells. (**A**) Expression of a mCherry fused human RNaseH1 in LN229 cells was verified by Western blot analysis using antibodies targeting mCherry and RNaseH1, as well as by intracellular mCherry signal. (**B**) Cellular growth of LN229 control (LN229-luc) and RNaseH1 expressing LN229 (LN229-R1) cells was determined by fluorescent intensity over 5 days. (**C**) Chromatin immunoprecipitation/qPCR experiments were performed using the S9.6 antibody at the 3’ end of the *ENSA*, *Gemin7* or *β-actin* genes in LN229-luc and LN229-R1 cells. Statistical analysis was done using Student’s *t*-test. **** = *p* < 0.0001. Detailed information about Western blot can be found at Figure S9.

To examine whether RNA:DNA hybrids could influence Ku70 binding at a DSB site, we produced LN229 cells overexpressing a mCherry fused human RNaseH1 (LN229-R1). RNaseH1 specifically degrades the RNA moiety of a RNA:DNA hybrid and is a common control in determining R-loop effects [8,27]. This mCherry-RNaseH1 fusion protein localizes to the nucleus due to a nuclear localization sequence. We confirmed RNaseH1 expression by performing Western blot analysis against RNaseH1 (Figure 3A). We observed three distinct bands when using a RNaseH1 specific antibody. As confirmation, a Western blot using a mCherry directed antibody was used. We found that the signal intensity of each mCherry band gradually decreased with lower molecular weight, suggesting that the three bands are formed by degradation of the mCherry tag (Figure 3A).

Expression of this exogenous RNaseH1 is confined to the nucleus as determined by immunofluorescence visualization (Figure 3A). As RNaseH1 can also localize to the mitochondria, we used a mitochondria specific antibody [28,29], to examine whether the exogenous protein was localizing to these structures and observed no colocalization of mCherry RNaseH1 with the mitochondria (Figure S8A). There was also no difference in cell growth (Figure 3B) or cell-cycle distribution (Figure S8B) between LN229-R1 and LN229-luc cells. Using the S9.6 antibody, we found a dramatic decrease in RNA:DNA hybrid formation at the 3’ end of the *ENSA*, *β-actin* and *Gemin7* genes in LN229-R1 cells as compared to LN229-luc cells (Figure 3C). These data suggest that the human RNaseH1 expressed in LN229 cells is functional and can resolve R loops.

**Figure 4:**
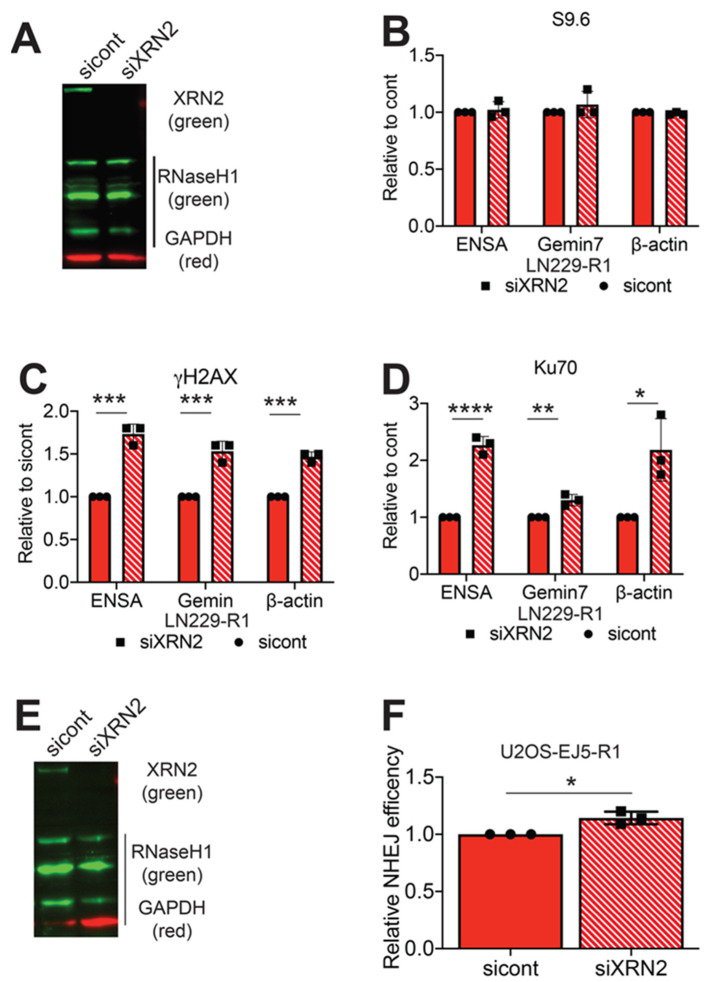
RNaseH1 expression restores NHEJ repair in XRN2 deficient cells. (**A**) Steady-state protein levels of XRN2 was measured in LN229-R1 cells exposed to control or XRN2 siRNAs. Chromatin immunoprecipitation/qPCR experiments were performed using (**B**) S9.6, (**C**) γH2AX and (**D**) Ku70 antibodies at the 3’ end of the *ENSA*, *Gemin7* and *β-actin* genes in LN229-R1 cells exposed to control and XRN2 siRNAs. (**E**) Steady state protein levels of XRN2 was determined in U2OS-EJ5-R1 cells exposed to control and XRN2 siRNAs. (**F**) Efficiency of NHEJ repair in the U2OS-EJ5-R1 reporter cells transfected with control or XRN2 siRNAs was determined by measuring 1000 cells for GPF expression and normalizing to control cells. Statistical analysis was done using Student’s *t*-test. * = *p* < 0.05, ** = *p* < 0.01 and **** = *p* < 0.00001. Detailed information about Western blot can be found at Figure S9.

After validating RNaseH1 function in LN229 cells, we examined the effect of RNaseH1 expression on Ku70 binding at the 3’ ends of the *β-actin, ENSA and Gemin7* genes. We transfected LN229-R1 with control or XRN2 siRNAs (Figure 4A) and performed ChIP/qPCR for Ku70. First, we examined for the presence of RNA:DNA hybrids at the 3’ end of the targeted genes and found no induction of S9.6 signal at any of these genomic loci after XRN2 loss (Figure 4B). Importantly, loss of XRN2 also lead to increases in γH2AX qPCR signals at all loci test in LN229-R1 cells, but to a lesser extent than the increases observed in LN229-luc cells (Figure 4C). Unlike LN229-luc cells, where there was a dramatic decrease in the amount of Ku70 accumulation at the 3’ end of the *ENSA*, *Gemin7* and *β-actin* genes after XRN2, we observed an increase in Ku70 signal at these three genomic loci after XRN2 loss (Figure 4D). To examine if RNaseH1 expression affects NHEJ repair, we generated EJ5 cells overexpressing RNaseH1 (EJ5-R1) (Figure 4E). To ensure that our results would not be affected by a change in the cell-cycle due to RNaseH1 expression, we compared the cell cycle distribution in U2OS-EJ5 and U2OS-EJ5-R1 cells. Similar to LN229 cells, we found no difference in the cell-cycle distribution between EJ5 and EJ5-R1 cells (Figure S8B). We exposed EJ5-R1 cells to control or XRN2 siRNAs (Figure 4F) and found no statistical difference in NHEJ repair efficiency (Figure 4E). Therefore, the presence of a RNA:DNA hybrid at the DSB site is detrimental to Ku70 binding and NHEJ repair.

### 2.5. RNaseH1 Expression Does Not Restore HR Repair

**Figure 5:**
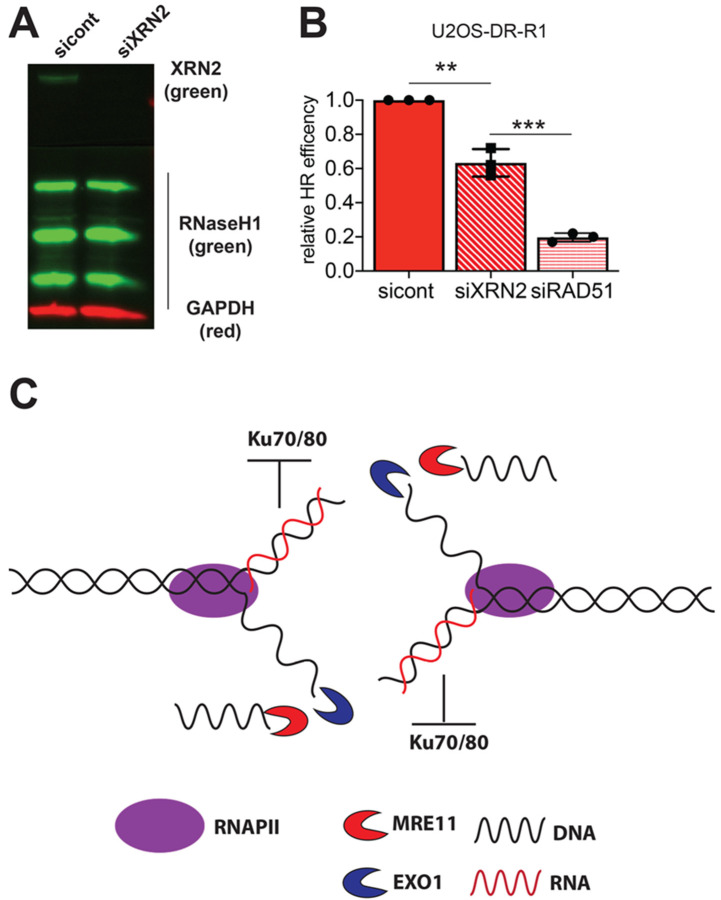
RNaseH1 expression does not rescue HR repair after XRN2 loss. (**A**) Steady-state protein levels of XRN2 was measured in U2OS-DR-R1 cells treated with control and XRN2 siRNAs. (**B**) Efficiency of HR repair in the U2OS-DR-R1 reporter cells exposed to control, XRN2 or Rad51 siRNAs was determined by measuring 1000 cells for GPF expression and normalizing to control cells. (**C**) Model of DSB repair in XRN2 deficient cells. Statistical analysis was done using Student’s *t*-test. ** = *p* < 0.01 and **** = *p* < 0.00001. Detailed information about Western blot can be found at Figure S9.

We examined whether RNA:DNA hybrid removal would restore HR repair capability after XRN2 loss. We generated human RNaseH1 overexpressing U2OS-DR cells (DR-R1) and confirmed expression of RNaseH1 by Western blot (Figure 5A). We exposed DR-R1 cells to control or XRN2 siRNAs and, unlike NHEJ repair, we observed a 40% decrease in GFP expressing cells after XRN2 loss in U2OS-DR-R1 cells (Figure 5B). Again, as in U2OS-DR, loss of RAD51 abolished all HR repair events (Figure 5B). Thus, RNA:DNA hybrid resolution at a DSB site though required [11], is not sufficient to restore HR repair.

## 3. Discussion

Repairing DSBs in a timely and efficient manner is an essential component for cellular survival. The cell typically uses one of two major cellular pathways to repair DSBs: HR and NHEJ [1]. The determining factors on DSB repair pathway choice are not fully understood. To complicate DSB repair, it has been found that a second highly regulated process, transcription, occurs at DSBs sites [11]. One of the consequences of transcription is the formation of a RNA:DNA hybrid at the DSB site [11]. Given these observations, we must now consider how regulating transcription and RNA:DNA hybrid formation at the DSBs affects DNA repair. One factor that functions in both transcription and DNA repair is XRN2 [8]. Our study indicates that XRN2 influences HR and NHEJ repair pathway choice through RNA:DNA hybrid removal.

### 3.1. Mechanistic Function of XRN2 in NHEJ Repair

We previously published that loss of XRN2 adversely affects NHEJ repair of a DNA plasmid substrate [8]. How loss of XRN2 leads to this decrease has remained a mystery. This current study suggests that the XRN2 resolves RNA:DNA hybrids formed at the DSB site and allows NHEJ to proceed. We demonstrate that while loss of XRN2 leads to an increase in γH2AX signal, a marker for DSB formation, there was a decrease in Ku70 at these DSB sites (Figure 1). Interestingly, we found a decrease in the amount of GFP expressing cells using the U2OS-EJ5 NHEJ reporter cells (Figure 1), which is consistent with our previous data showing that XRN2 loss inhibits repair of a DNA plasmid substrate [8]. Our result is similar to that seen when preforming the NHEJ assay in DNAPK or XRCC4 deficient MEFs [30], which is consistent with our observation that loss of XRN2 results in decreased amounts of activated DNAPKcs (Figure S1). The fact that overexpression of RNaseH1 restores Ku70 binding to DNA ends and NHEJ repair capability, denotes that the presence of a RNA:DNA hybrid plays a role in mediating NHEJ repair by inhibiting the initial steps of the repair process.

Interestingly, recent yeast studies demonstrated that loss of the RNA:DNA helicase Sen1 leads to RNA:DNA hybrid formation after a DSB is generated by the HO endonuclease and the formation of this hybrid resulted in accumulation of yKu70 at DSB site [31]. While these data appear to be contradictory to the data we presented, there are important considerations to take mind of. First, although possible, it is highly improbable that Ku70 is interacting with the RNA:DNA hybrid per se. Crystal structure data reveals that the Ku70/80 dimer fits precisely around double stranded DNA (dsDNA) [32]. Although the Ku70/80 heterodimer can interact with RNA, this interaction requires that the RNA have a stem-loop secondary structure [33]. Ku70/80 does not recognize double stranded RNA without the stem-loop structure [33]. An RNA:DNA hybrid is an intermediate between B-form double stranded DNA and A-form double stranded RNA, with an overall structure more similar to double stranded RNA than double stranded DNA [34]. In the absence of the required RNA secondary structure, an RNA:DNA hybrid would inhibit Ku70/80 binding due to the fact that the hybrid is larger and has a severely different overall structure when compared to a DNA:DNA complex [35].

A second consideration is the presence of PARP1. PARP1 competes with and actively removes the Ku70/80 dimer from DSB sites. Yeast have no endogenous PARP1 activity [36]. We demonstrate that cells lacking XRN2 display increased PARP1 activation (Figure S6C) and overly rely on PARP1 for survival (Figure S6B). PARP1 has also been shown to interact with and function in RNA:DNA hybrid DNA damage [37,38]. In addition, PARP1 is involved in the removal of an RNA moiety for the DNA template [39]. Thus, the presence of PARP1 can explain the loss of Ku70 at DSB sites. PARP1 activity may also explain why we find an accumulation of HR related repair factors, such as BRCA1, CtIP and EXO1 (Figure 5) at DSB sites with loss of XRN2 [8]. Therefore, the R loop acts as a physical barrier preventing the initiation of cNHEJ repair.

### 3.2. XRN2 Loss Inhibits HR Repair Due to Excessive DNA End Resection

One novel discovery made in this study is a role for XRN2 in mediating HR repair. Previously, we found that loss of XRN2 led to an increase in both chromosome and chromatid type breaks, which primarily results from defects in NHEJ and HR repair, respectively [8]. We also found that loss of XRN2 resulted in defects in NHEJ repair [8], which we mechanistically describe in this manuscript. However, the role of XRN2 in HR repair has been unexplored in previous efforts.

Using the U2OS-DR reporter cells, that measure HR repair [15], we found that loss of XRN2 lead to a decrease in HR capabilities (Figure 2A). XRN2 loss also led to a decrease of RPA32 binding at a DSB sites (Figure 2B). This evidence would suggest that RNA:DNA hybrids are responsible for the decrease in HR repair by inhibiting RPA binding at the DSB site. However, when we removed the influence of RNA:DNA hybrids by the overexpression of RNaseH1, HR repair capability was not restored. Therefore, RNA:DNA hybrid removal is not the reason for the HR defects observed in XRN2 lacking cells. Given the fact that we observed an increase in CtIP at the 3’end of the *β-actin* gene [8], we examined whether DNA end degradation was properly regulated, as CtIP helps to regulate this process [40]. Indeed, we found excess amounts of EXO1 at the 3’ end of the *ENSA*, *Gemin7* and *β-actin* genes. In addition, accumulation of EXO1 and RNA:DNA hybrids were observed up- and downstream of the *β-actin* gene 3’ pause site.

One possible mechanism of XRN2 limiting end-resection is through transcription regulation. XRN2 mediates transcription termination [12] and RNAPII displacement from the DNA template at the 3’ end of the *β-actin* gene [13]. RNAPII transcription is a process that results in DNA unwinding and DNA:RNA hybrid formation. Our data suggest that while XRN2 does mediate hybrid removal, its major function in HR repair is to limit DNA end-resection, possibly through displacement of the RNAPII machinery from the DNA template, as excessive DNA end degradation at the DSB site is a major detriment to HR repair [26,41].

In summation, RNA:DNA hybrid removal does not restore HR repair and XRN2 loss leads to an excess amount of DNA end degradation. With the data we have presented, we propose a model where loss of XRN2 results in prolonged existence of a RNA:DNA hybrid at the DSB site, which in turn inhibits NHEJ repair by blocking the Ku70/80 heterodimer. MRE11 then processes the DSB end, facilitating EXO1 recruitment and allowing for excessive DNA end resection and generation of an extended single stranded DNA moiety which is detrimental to HR repair (Figure 5C).

One question that remains is if XRN2 mediated transcription termination a general mechanism of DSB repair or does it occur solely at the 3’ end of genes where termination is regulated by XRN2.

## 4. Materials and Methods

### 4.1. Colony Forming Assays

500 LN229-luc and LN229-shXRN2 cells were plated onto 60 mm tissue culture plates and allowed to grow for two days. Cells were then exposed to PARP1 inhibitor (at indicated doses), allowed to grow for 7 days, washed with PBS and stained with crystal violet solution. Colonies with > 50 normal appearing cells were counted, and percent survival was calculated and graphed with dose.

### 4.2. Cell Culture

LN229, G55, 293T and U2OS were maintained in 5% CO2 at 37 ℃. LN229 were cultured in 5% fetal bovine serum (FBS) in high glucose L-glutamine containing DMEM (DMEM) supplemented with 1× penicillin/streptomycin (Pen/Strep). G55, 293T and U2OS were cultured in 10% FBS in DMEM with 1× Pen/Strep.

### 4.3. Generation of Human RNaseH1 Cells

293T were forward co-transfected with RNaseH1-wildtype-mcherry and 3rd-generation lentiviral packaging plasmids using Sigma Universal Transfection reagent (Sigma Aldrich, St. Louis, MO, USA, cat no. T0956-1ML). Virus were harvested at 48 h, 72 h, 96 h, and 120 h post-transfection and supplemented with Polybrene. Recipient cell line were infected with viruses and selected with 1 µg/mL puromycin. Validation of exogenous expression was done via Western blot (RnaseH1 and mCherry) and fluorescence.

### 4.4. Western Blot

Immuno-blotting was carried out as previously described [42]. Briefly, cells were lysed in RIPA buffer and supplemented with protease inhibitors. After addition of Laemmli sample buffer, samples were run on a 10% SDS-Page gel. Samples were transferred onto a PVDF membrane via BioRad’s semi-dry apparatus. Membranes were blocked in 0.01% Casien block for 1 h and incubated with antibodies at 4 °C overnight (O/N). Membranes were then washed with TBST buffer, probed with 2° antibodies, washed with TBST buffer and then imaged on a BioRad Chemidoc MP. See Table S1 for a list of antibodies used.

### 4.5. Immunofluorescence (IF)

IF was carried out as previously described [42]. Cells were fixed with 2% formaldehyde in PBS and permeabilized with 0.5% Triton X-100. Cells were blocked with 10% serum in immunofluorescence buffer (IF buffer) (PBS, 1% BSA, 0.2% Triton X-100 and 0.05% Tween-20) for 1 h and the incubated with antibodies O/N. Samples were washed 3× in IF buffer, probed with 2° antibodies, washed 3× in IF buffer and stored in PBS at 4 °C until imaging. See Table S1 for a list of antibodies used. XRN2 IF was performed as previously described [8].

### 4.6. I-SecI-Based DNA Repair Assays

Assays were carried out as described with slight modifications [15]. Cells were reverse transfected with 50 nM of siRNA and siRNA transfection reagent (Sigma Aldrich, cat no. S1452-1ML) in a 96-well plate for 24 h, then I-SecI plasmid (60 ng/well) was transfected into the cells, no additional amount of siRNA needed. Media was replaced 24 h after plasmid transfection and cells were fixed with 2% formaldehyde in PBS and counter stained with Hoechst 72 h post-plasmid transfection. Cells were imaged for Hoechst, GFP and mCherry signals. Percent efficiency was determined by percent cells positive for GFP and Hoechst (EJ5-GFP and DR-GFP) or for GFP and mCherry (EJ5-R1 and DR-R1).

### 4.7. Imaging

Imaging was done on a BioTek’s Cytation 5 imager. The following objectives were used 10× phase Plan Fluorite WD 10 NA 0.3 and 20× phase Plan Fluorite WD 6.7 NA 0.45. Spot counting is a propriety application within Gen5 (BioTek, Winooski, VT, USA).

### 4.8. Chromatin Immuno-Precipitation/qPCR (ChIP/qPCR)

ChIP was performed as previously described [43]. Briefly, cells were fixed in 0.1% formaldehyde in media for 15 min, quenched at a final concentration of 0.125 M glycine, incubated with PIPES hypotonic buffer for 30 min at 4 °C, and then lysed in RIPA buffer at 4 °C O/N with rotation. Samples were sonicated 30 s ON/OFF on high for a total time of 25 min on a Diagene Bioruptor. Each pulldown had 500 µg of chromatin, 2–5 µg of antibody, and 50 µL of Protein A/G Plus Agarose (Thermofisher, Waltham, MA, USA, cat no. 20424) incubated at 4 °C O/N with rotation. Pulldowns were incubated in ChIP Wash Buffers 1–4 for 10 min at 4 °C. Pulldowns were pelleted and resuspend in 10 µg RNaseA and 10 µg Proteinase K in Resuspension Buffer. Samples were incubated at 55 °C for 3 h, 65 °C ~16 h and then cleaned up with QIAquick PCR purification kit (Qiagen, cat no. 28106). PCR was conducted using iTaq Universal SYBR Green Supermix (BioRad, Hercules, CA, USA, cat no. 1725125) on a BioRad CFX96 Real-Time System. See Supplemental Materials for PCR primer sequences.

### 4.9. Statistics

All experiments (including Western blots and immunofluorescence images) were performed three or more times. Means and standard errors were calculated and differences between treatments were determined by confidence limit calculations using Student’s *t*-tests. *p* values (0.01 and 0.05) for 99% and 95% confidence limits, respectively, were considered significant and reported.

## 5. Conclusions

In conclusion, our study reveals a previously unreported role for RNA:DNA hybrid resolution in regulating the decision on how DSB are repaired. In terms of cancer this is an important finding due to the fact that RNA:DNA hybrids are a major sources DSB breaks tumors. It has been shown that RNA:DNA hybrids are responsible for DSB formation in estrogen induced DNA damage [44]. RNA:DNA hybrids have also been shown to be responsible for dangerous translocations that could lead to tumor formation [45,46]. Thus, understanding how these structures are regulated in response to DNA damage and at the DSB site is of upmost importance to preventing cancer disease states.

## Supplementary Materials

The following are available online at https://www.mdpi.com/2072-6694/12/7/1821/s1, Figure S1: XRN2 colocalizes with 53BP1 after IR, Figure S2: Loss of XRN2 decreases DNAPK-pT-2609 phosphorylation, Figure S3: Confirmation of XRN2 loss in LN229-luc cells, Figure S4: Cell cycle distribution of LN229 cell with and without XRN2, Figure S5: Loss of XRN2 impairs Ku70 binding to 3’ pause site of the β-*actin* gene, Figure S6: Loss of XRN2 does not affect aNHEJ and sensitizes cells to PARP1 inhibition, Figure S7: Loss of XRN2 results in increased spontaneous MRE11 foci formation, Figure S8: Ectopically expressed RNaseH1 does not colocalize to mitochondria or alter the cell cycle in LN229, U2OS-EJ5 or U2OS-DR cells, Figure S9: Detailed information about western blot, Table S1: Plasmid, antibodies and siRNA sequences.
