# Supplementary material for: XRN2 Links RNA:DNA Hybrid Resolution to Double Strand Break Repair Pathway Choice
Source: Cancers (Basel). 2020 Jul 7;12(7):1821. doi: 10.3390/cancers12071821 (PMC7408924; doi:10.3390/cancers12071821)
Supplement: Supplementary file 1 [file cancers-12-01821-s001.pdf]

*Supplementary Materials*

# XRN2 Links RNA:DNA Hybrid Resolution to Double Strand Break Repair Pathway Choice

**Tuyen T. Dang and Julio C. Morales**

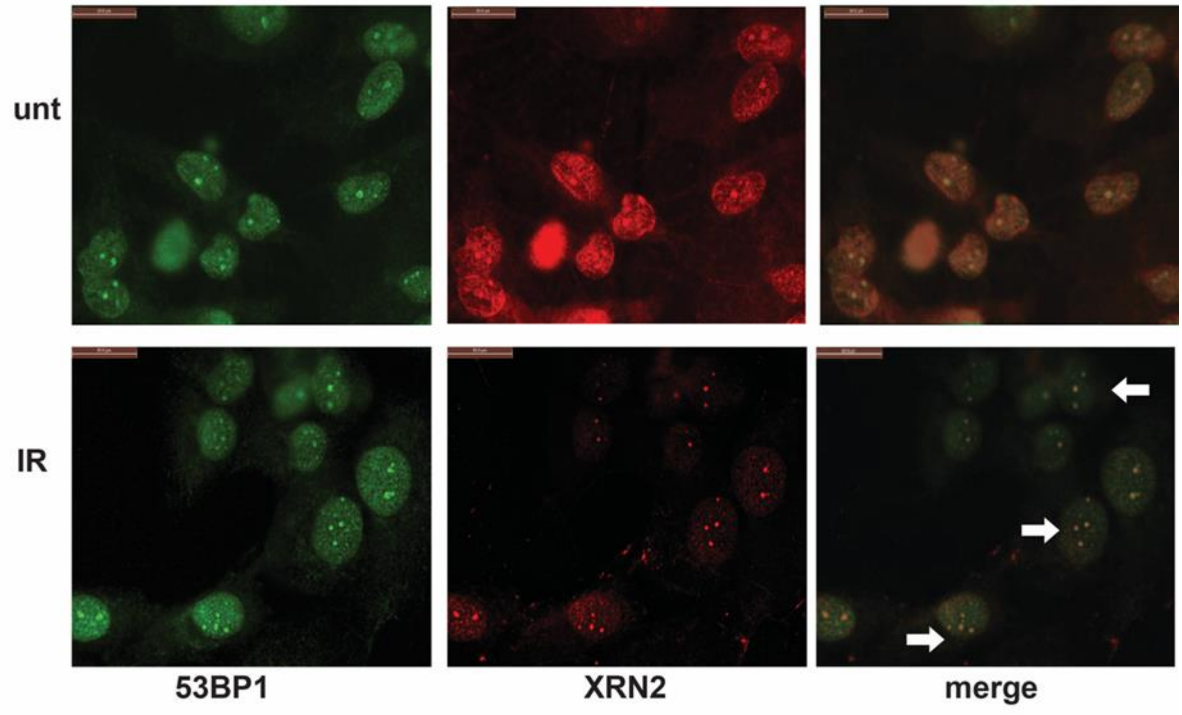


**Figure S1.** XRN2 colocalizes with 53BP1 after IR. Previously described immortalized human fibroblast (Morales et al. 2016), were used to visualize 53BP1 and XRN2 foci. Cells were either mock or IR (0.5 Gy) treated. White arrows depict examples of 53BP1 and XRN2 colocalization.

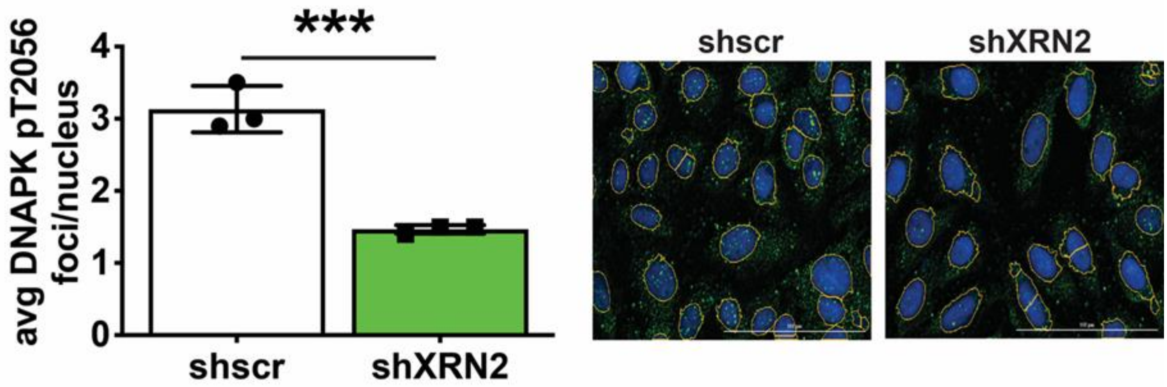


**Figure S2.** Loss of XRN2 decreases DNAPK-pT-2609 phosphorylation. Previously described immortalized human fibroblast (Morales et al. 2016), were used to visualize spontaneous DNAPK-pT-2609 foci formation, as visualized using a DNAPK-pT-2609 specific antibody. Experiments were performed in triplicate and 100 cells were counted for each condition in each experiment. Statistical analysis was performed using a student's *t* test. *** = $p$ <.001.

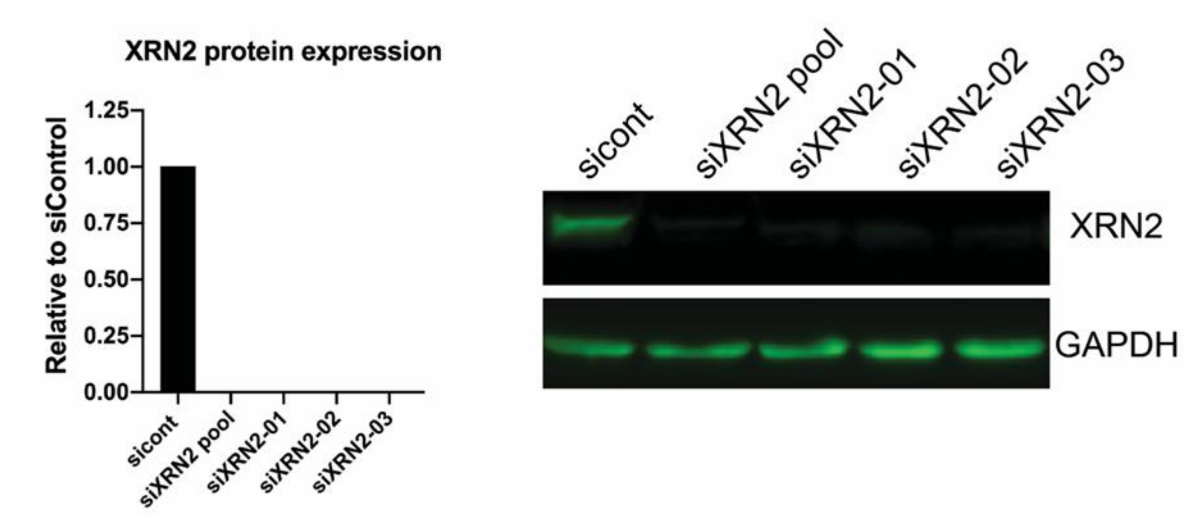


**Figure S3.** Confirmation of XRN2 loss in LN229-luc cells. Steady state protein levels of XRN2 was measured in LN229-luc cells treated with control, pooled or one of three non-overlapping XRN2 siRNAs. Relative protein expression was measure by using the BioRad Chemidoc MP. Detailed information about western blot can be found at Figure S9.

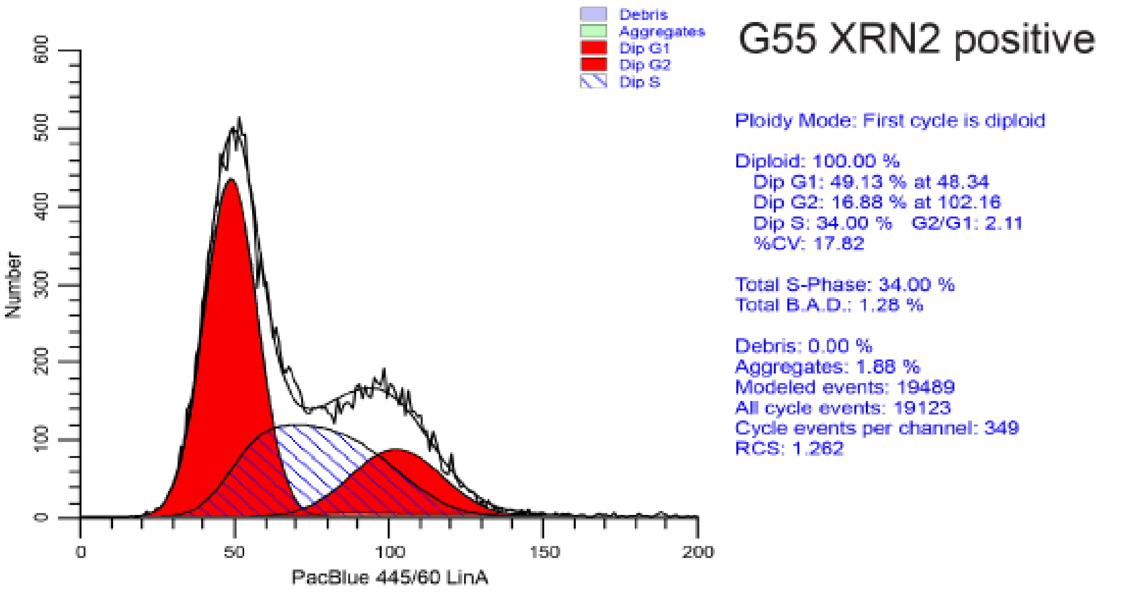


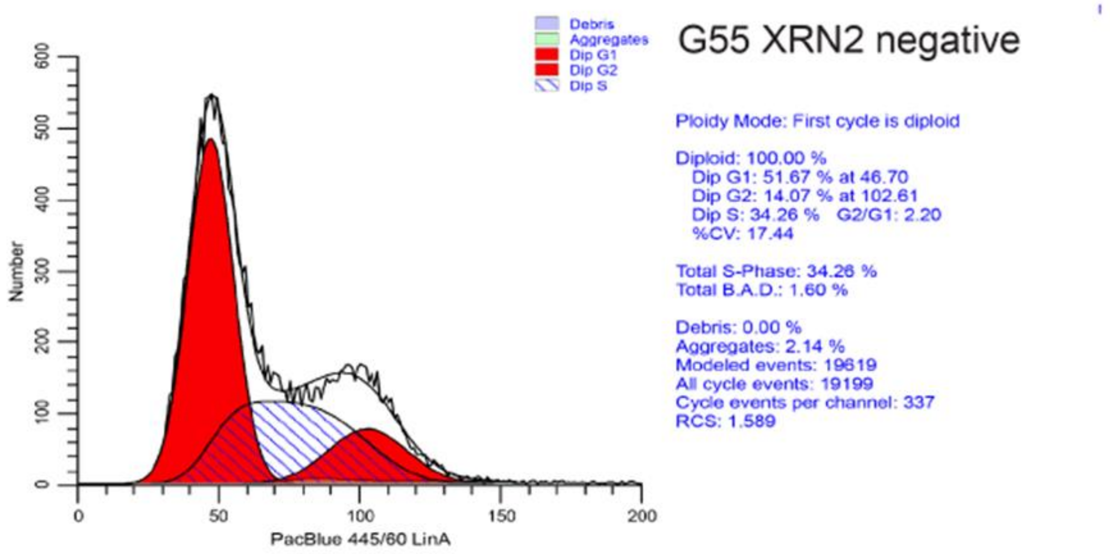


**Figure S4.** Cell cycle distribution of LN229 cell with and without XRN2. The cell cycle distribution of asynchronously growing LN229 cells with and without XRN2 was measured by DAPI incorporation.

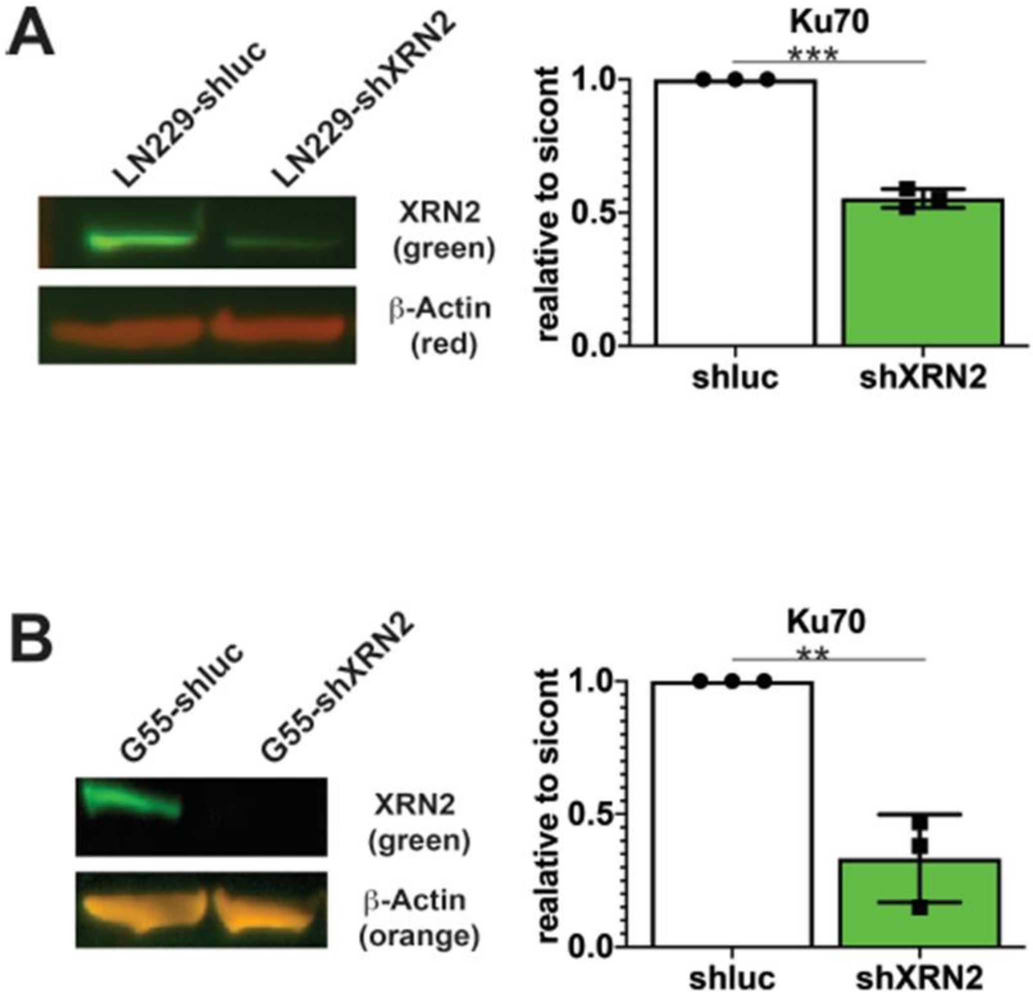


**Figure S5.** Loss of XRN2 impairs Ku70 binding to 3′ pause site of the β-*actin* gene. (**A,B**) Steady state protein levels of XRN2 protein was determined by western blot in G55 and LN229 cells with and without XRN2. Chromatin immunoprecipitation/qPCR experiments were performed using a Ku70 antibody in (**A**) LN229 control (shluc) and XRN2 deficient (shXRN2) and (**B**) G55 control (shluc) and XRN2 deficient (shXRN2) cells. Statistical analysis was performed using a student's *t*-test. *** = $\mathbf{p} < 0.001$ and ** = $p < 0.01$. Detailed information about western blot can be found at Figure S9.

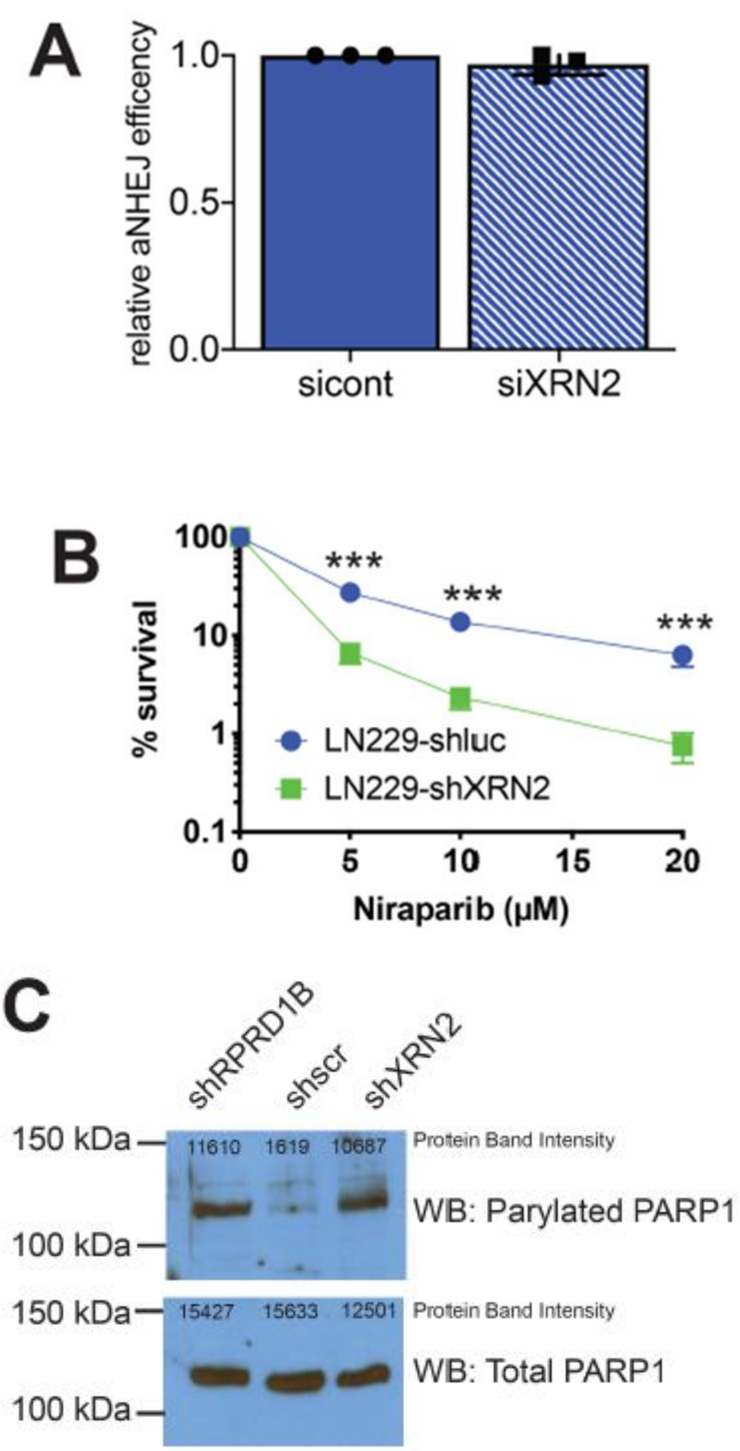


**Figure S6.** Loss of XRN2 does not affect aNHEJ and sensitizes cells to PARP1 inhibition**.** (**A**) Efficiency of aNHEJ repair in the U2OS-EJ2 reporter cells exposed to control or XRN2 siRNAs was determined by measuring 1000 cells for GFP expression. (**B**) Colony forming ability was measured in control (LN229-shluc) and XRN2 lacking (LN229-shXNR2) cells exposed to the PARP1 inhibitor Niraparib (Jones et al. 2009) at indicated doses. (**C**) Steady state protein levels of PARP1 and parylated PARP1 were measured in control, shRPRD1B (Morales et al. 2014) and shXRN2 (Morales et al. 2016) cells. Protein band intensities were determined using NIH ImageJ.Statistical analysis was done using a student's t test. *** = $p < 0.0001$.

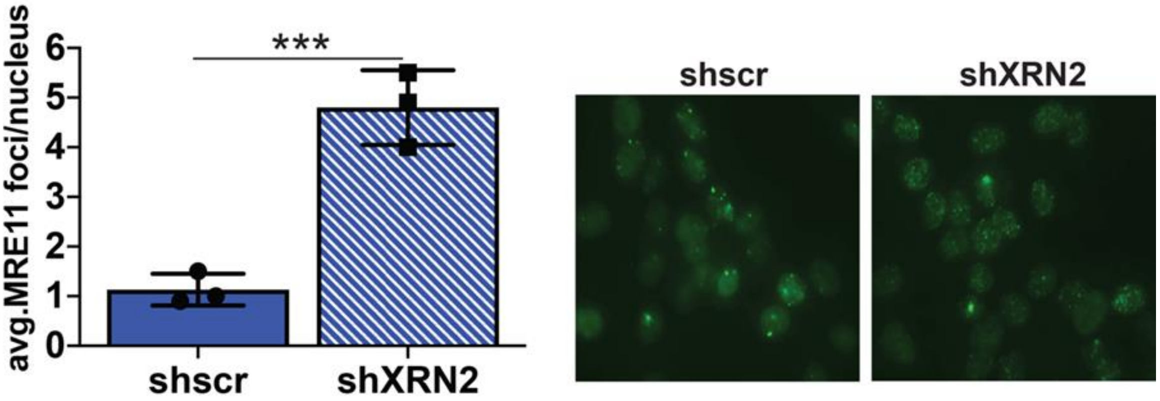


**Figure S7.** Loss of XRN2 results in increased spontaneous MRE11 foci formation**.** Previously described immortalized human fibroblast with (shscr) and without XRN2 (shXRN2) (Morales et al. 2016) were used to visualized spontaneous MRE11 foci formation. Experiments were performed in triplicate and 100 cells were counted in each experiment. Statistical analysis was done using a student's *t* test. *** = $p < 0.0001$. Images taken at 63× magnification

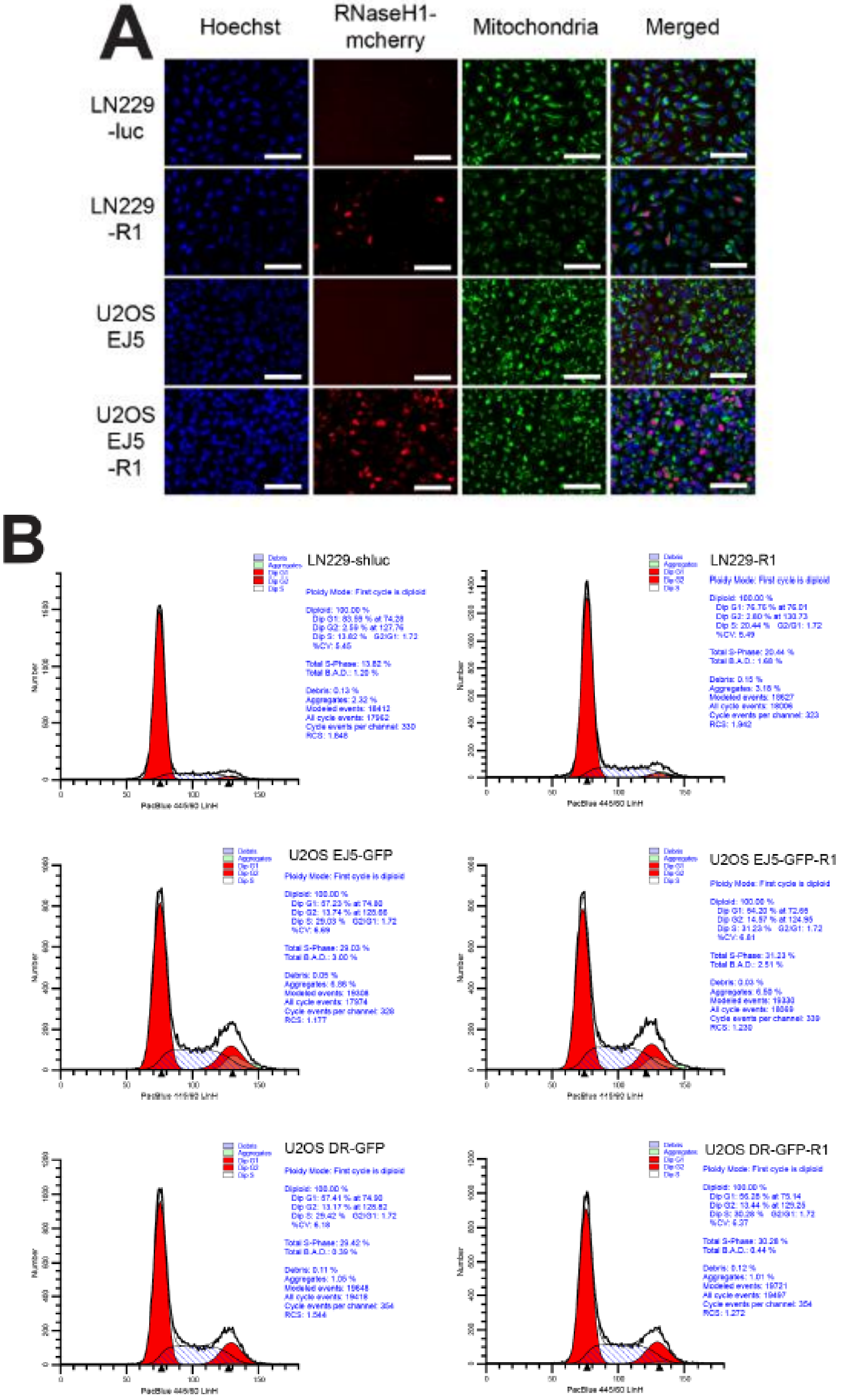


**Figure S8.** Ectopically expressed RNaseH1 does not colocalize to mitochondria or alter the cell cycle in LN229, U2OS-EJ5 or U2OS-DR cells. (**A**) A mitochondria specific antibody, (green) was used to label the mitochondria in LN229-luc, LN229-R1, U2OS-EJ5 and U2OS-EJ5-R1 cells. (**B**) The cell cycle distribution of asynchronously growing LN229-luc, LN229-R1, U2OS-EJ5-luc and U2OS-EJ5-R1 cells was measured by DAPI incorporation. White scale bar = 100 μm.

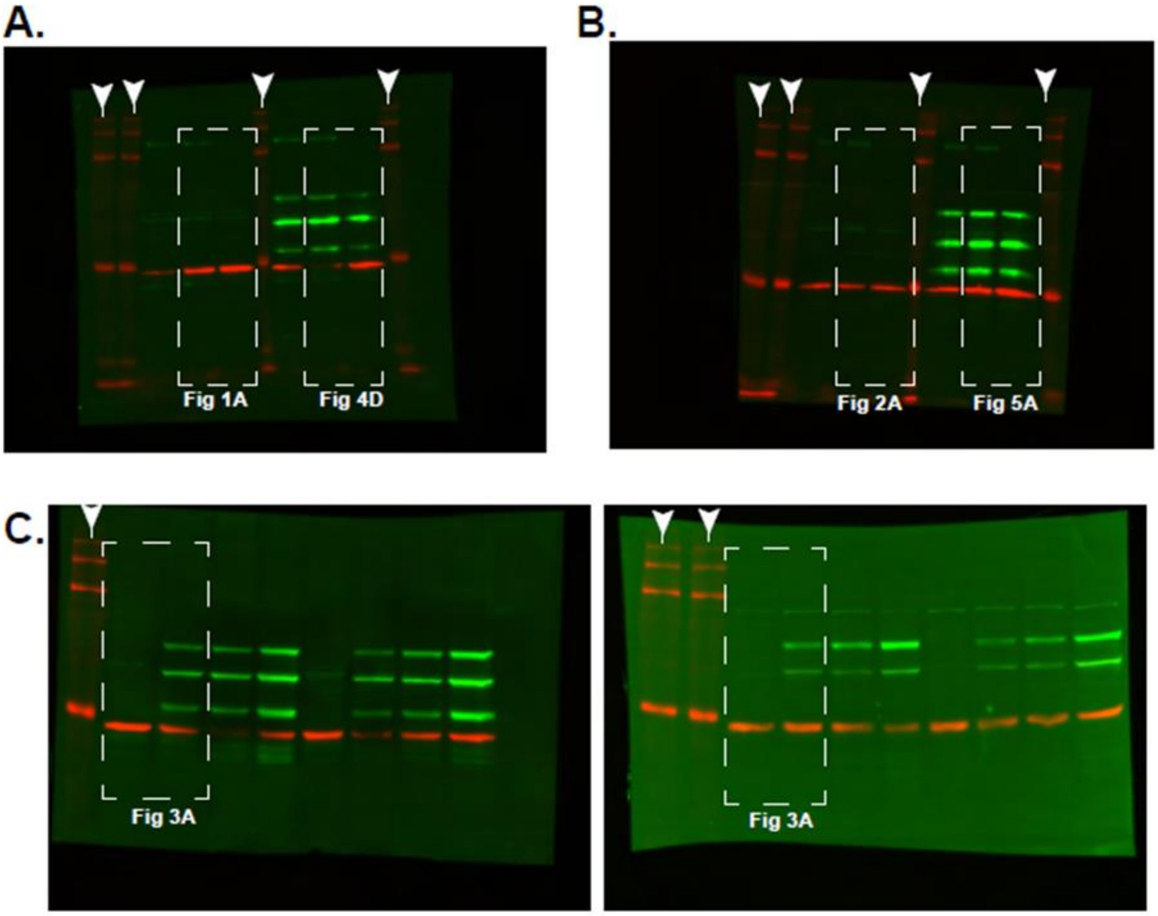


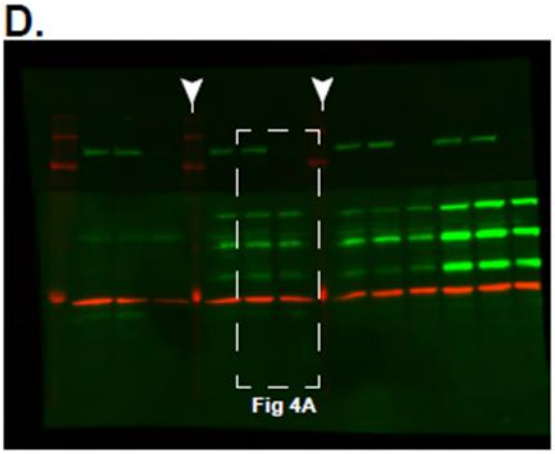


Dotted rectangle specifies the portion of the blot that was used for the figures. Arrows mark the protein ladder (BioRad, cat. no. 161-0376).

E.

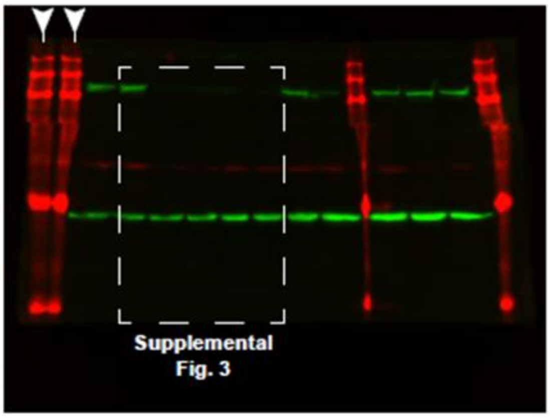


| | | | |
|---|---|---|---|
| XRN2 expression | | | |
| Channel | Sample | Label | Adj. Vol. (Int) |
| Alexa 488 | sicont | U2 | 12,554,663.21 |
| | siXRN2 total | x | 0 |
| | siXRN2-01 | x | 0 |
| | siXRN2-02 | x | 0 |
| | siXRN2-03 | x | 0 |
| GAPDH expression | | | |
| Channel | Sample | Label | Adj. Vol. (Int) |
| Alexa 488 | sicont | U9 | 15,766,477.40 |
| Alexa 488 | siXRN2 total | U10 | 15,867,737.70 |
| Alexa 488 | siXRN2-01 | U11 | 18,381,514.11 |
| Alexa 488 | siXRN2-02 | U12 | 23,787,639.78 |
| Alexa 488 | siXRN2-03 | U13 | 21,058,681.88 |

F.

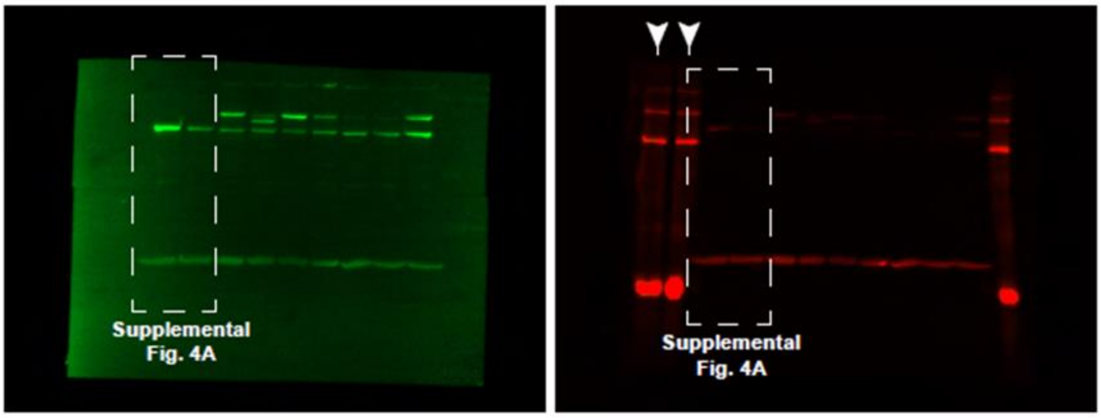


G.

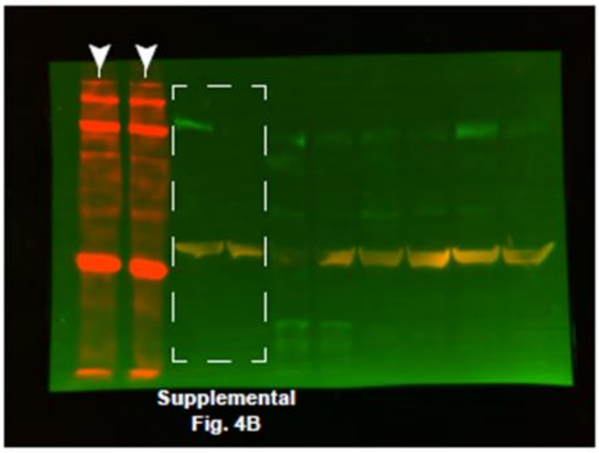


Dotted rectangle specifies the portion of the blot that was used for the figures. Arrows mark the protein ladder (BioRad, cat. no. 161-0376).

**Figure S9.** Detailed information about western blot.

**Table S1.** Plasmid, antibodies and siRNA sequences.

| **Plasmids** | | | |
|---|---|---|---|
| **Name** | **Vendor** | **Catalog Number** | |
| RNaseH1 | GeneCopeia | EX-A5399-Lv130 | |
| pMDLg/pRRE | Addgene | 12251 | |
| pMD2.G | Addgene | 12259 | |
| pRSV-Rev | Addgene | 12253 | |
| shRNAseH1 | Sigma Aldrich | SHCLNG, TRCN0000331261 | |
| pCBASceI | Addgene | 26477 | |
| pCAGGS-I-SceI-Trex2 | Addgene | 44024 | |
| **Antibodies** | | | |
| **Name** | **Vendor** | **Catalog Number** | |
| XRN2 | Bethyl | A301-103A | |
| 53bp1 | ThermoFisher | PA1-16566 | |
| beta Actin | ThermoFisher | MA5-15739 | |
| gH2AX | Millipore | 05636 | |
| GAPDH | Abcam | ab181602 | |
| mCherry [1C51] | Abcam | ab125096 | |
| RNaseH1 | Abcam | ab56560 | |
| S9.6 | | Gift from | |
| IgG | Santa Cruz | sc2025 | |
| Ku70 | ThermoFisher | MA5-13110 | |
| Anti-Replication Protein A Antibody, clone RPA34-20 | Sigma | MABE285 | |
| **SiRNAs** | | | |
| **Name** | **Vendor** | **Sequence** | **Catalog Number** |
| SASI_Hs01_00190258, XRN2 | Sigma Aldrich | GAGUACAGAUCAUGUU | |
| SASI_Hs01_00190257, XRN2 | Sigma Aldrich | CAUCGUUAGAGAUUAGGGA | |
| SASI_Hs01_00190260, XRN2 | Sigma Aldrich | CGAUAGUCUUCCUUGUGCA | |
| SASI_Hs01_00201423, UBB | Sigma Aldrich | GCACUCUUUCUGACUACAA | |
| SASI_Hs01_00201424, UBB | Sigma Aldrich | GCCAAGAUCCAAGAUAAAG | |
| SASI_Hs01_00201425, UBB | Sigma Aldrich | GUACUCUUUCUGACUACAA | |
| ON-Targetplus Non-targeting pool | Dharmacon | UGGUUUACAUGUCGACUAA, UGGUUUACAUGUUGUGUGA, UGGUUUACAUGUUUUCUGA, UGGUUUACAUGUUUUCCUA | D-001810-10-05 |

© 2020 by the authors. Licensee MDPI, Basel, Switzerland. This article is an open access article distributed under the terms and conditions of the Creative Commons Attribution (CC BY) license (http://creativecommons.org/licenses/by/4.0/).
